# Spiral Form of the Human Cochlea Results from Spatial Constraints

**DOI:** 10.1038/s41598-017-07795-4

**Published:** 2017-08-08

**Authors:** M. Pietsch, L. Aguirre Dávila, P. Erfurt, E. Avci, T. Lenarz, A. Kral

**Affiliations:** 1 Institute of AudioNeuroTechnology & Dept. of Experimental Otology, ENT Clinics, School of Medicine, Hanover Medical University, Hanover, Germany; 2 Institute of Biometry, School of Medicine, Hanover Medical University, Hanover, Germany; 3 School of Behavioral and Brain Sciences, The University of Texas, Dallas, USA

## Abstract

The human inner ear has an intricate spiral shape often compared to shells of mollusks, particularly to the nautilus shell. It has inspired many functional hearing theories. The reasons for this complex geometry remain unresolved. We digitized 138 human cochleae at microscopic resolution and observed an astonishing interindividual variability in the shape. A 3D analytical cochlear model was developed that fits the analyzed data with high precision. The cochlear geometry neither matched a proposed function, namely sound focusing similar to a whispering gallery, nor did it have the form of a nautilus. Instead, the innate cochlear blueprint and its actual ontogenetic variants were determined by spatial constraints and resulted from an efficient packing of the cochlear duct within the petrous bone. The analytical model predicts well the individual 3D cochlear geometry from few clinical measures and represents a clinical tool for an individualized approach to neurosensory restoration with cochlear implants.

## Introduction

Living creatures and their organs are often intricate, complex and delicate in form. The form-to-function relation has been an important question of biology since centuries, in particular focus after the seminal work of D’Arcy Thompson^1^. In Thompson’s view, there is a formative power of physical (and biological) forces that shapes the appearance of an organism and can be revealed by mathematical analysis of its form. Mathematical description of complex forms can thus provide information for the underlying principle that shaped its growth^1^. Complex natural forms can sometimes be explained by simple underlying causes and may follow simple mathematical relations, e.g. the ‘sectio aurea’^2–5^. Biological forms are, in their blueprint, defined by the genetic makeup, but can be phylogenetically or ontogenetically shaped by a function or by biological interactions with surrounding structures. Function limits the interindividual (ontogenetic) variation of the form in those measures that are critical for the function.

Inner ear geometry has been traditionally compared to shells of mollusks, as the name ‘cochlea’ documents. Due to the apparent similarity to the cochlea, the nautilus shell has become a favorite symbol of hearing^1,6,7^. The nautilus shell is a perfect biological example of a logarithmic Fibonacci spiral^8^. Correspondingly, logarithmic is also the relation between characteristic (best) frequency of auditory nerve fibers and the cochlear position they innervate^9–11^. What kind of geometrical spiral the cochlea really follows has, however, not been mathematically analyzed yet.

Also the reasons for the spiral form of the mammalian inner ear remain unclear. The efficient packing hypothesis assumes the shape of the inner ear to be the consequence of spatial restrictions within the petrous bone^12–15^. The efficient packing hypothesis involves a strong influence of local factors in the morphogenesis. The stronger the influence of such local factors, the more likely are individual variations in morphology. On the other hand, historically, the cochlear shape also inspired many functional hearing theories. Recently it has been suggested that, because of its shape, the inner ear functions as a whispering gallery focusing low-frequency sounds to the apex of the cochlea^16–19^. In order to follow a function, interindividual variability of the corresponding critical aspects of the shape would have to be minimal.

Additional to the large variability in the cochlear morphology between different mammals^15^, there is interindividual variability in cochlear size in animals^15^ and humans^20–24^. Mathematical analysis of the cochlea has, thus far, only concentrated on the overall length^20,24–27^ or the distribution of primary afferents relative to cochleotopic organization^9,10^, but has rarely considered cochlear shape as such^6^.

**Figure 1:**
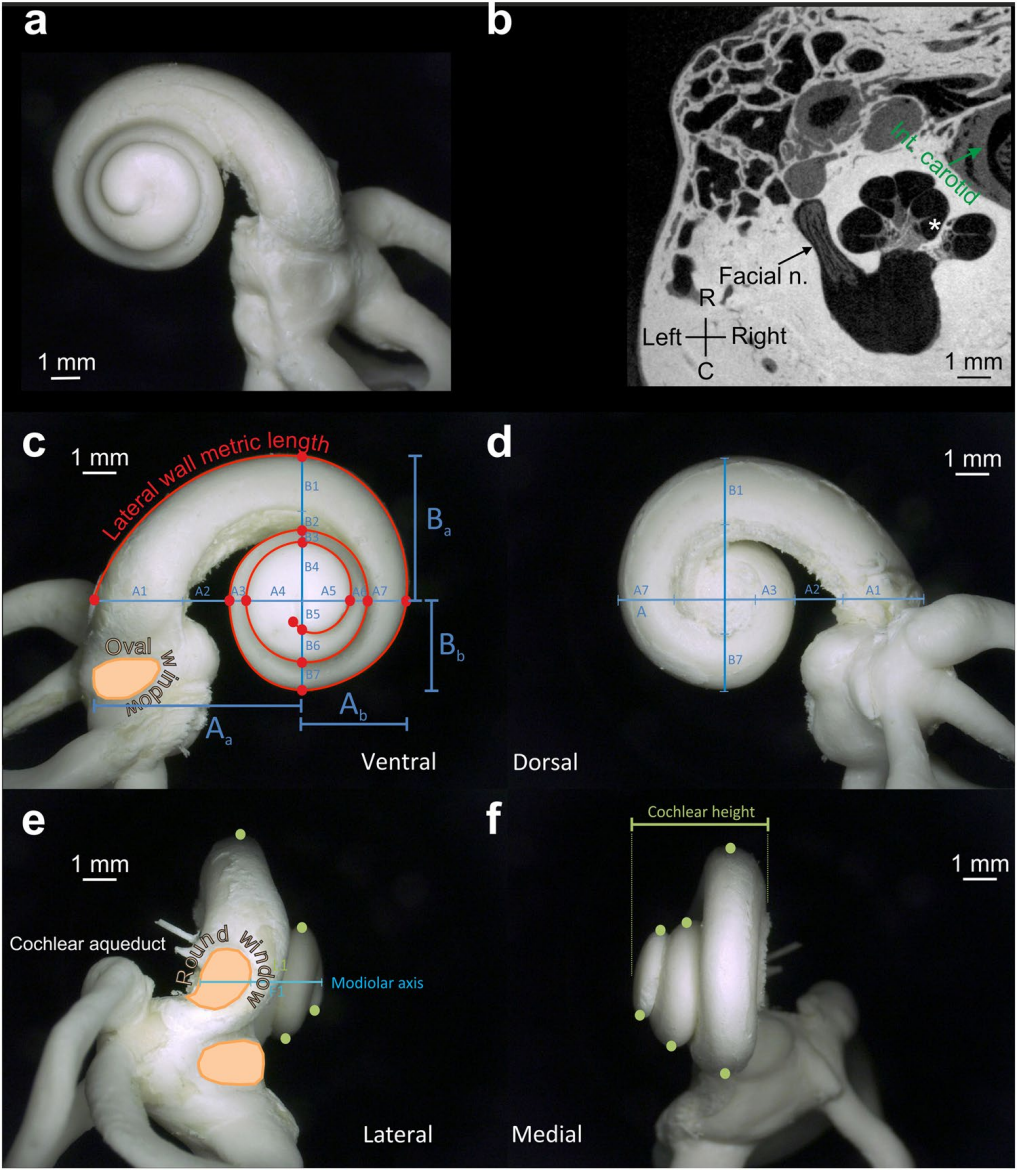
Quantification of cochlear geometry. (**a**) Corrosion cast of a human cochlea photographed through the microscope, view from the apex (rostral view). (**b**) Modiolar section of the human cochlea (µCT imaging). In addition to the cochlea, the facial nerve and the internal carotid artery are also visible in these images. Asterisk denotes the scala tympani of the second cochlear turn, R = rostral, C = caudal. (**c**) Rostral view of a corrosion cast and the main parameters (measuring points marked by red dots) further discussed in the present study. Oval window is discernible in this view (orange color). Red line marks the cochlear length along the lateral wall (metric length). Cochlear axes (A and B, sometimes called cochlear base length and width) were determined from the sum of 7 subcomponents. Their mutual intersection defines the parts A_a_ and A_b_ (and B_a_ and B_b_, respectively). (**d**) Caudal view of a corrosion cast and the parameters quantified in these photographs. These measures were validating the measures in (**c**) and the corrections for tilt of the modiolar axis. (**e**) Lateral view of the cochlea; the green dots mark the points determined for calculation of the height profile. These measures were further corrected for the individual deviation of the modiolar axis from 0°. Round and oval window are discernible in this view (orange colors). (**f**) Medial view of the cochlea; the green dots mark the points determined for calculation of the height profile. Cochlear height was the shortest distance between the apicalmost and basalmost point of the cochlea. All measurements were corrected for the individual deviation of the modiolar axis from 0°.

To understand the variations in cochlear geometry within one species and to allow personalized prediction of the individual cochlear form for neurosensory restoration using cochlear implants, we analyzed a set of adult human cochleae obtained from 108 individuals by means of the corrosion cast technique^22,23^ (Fig. 1a) and cochleae obtained from 30 individuals using µCT imaging (Fig. 1b). Corrosion cast images were digitized in three dimensions using a digital microscope (resolution of 12 µm per pixel) and a customized micromanipulator. Using these digitized images, 120 parameters characterizing the detailed form were measured in each cochlea (some shown in Fig. 1). The µCT scans of human cochleae^24^ verified the validity of the corrosion cast measurements and additionally allowed 3D reconstructions of the structures surrounding the cochlea that were not visible in corrosion casts.

## Results

**Figure 2:**
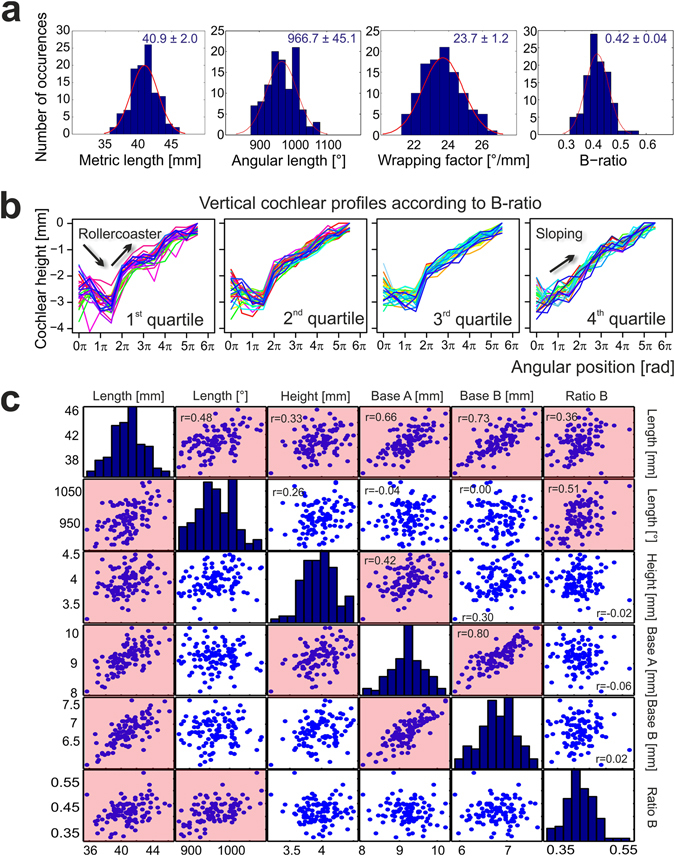
Large variance in the size and shape of the human cochlea. (**a**) Histograms of metric length, angular length, wrapping factor and B-ratio determined in the corrosion casts (B-ratio = B_a_/(B_a_ + B_b_)). The results demonstrate extensive interindividual variance in the cochlear geometry. (**b**) Vertical profiles of the uncoiled cochleae, i.e. height (relative to apex) versus angular position. All cochleae were divided into quartiles based on the B-ratio. In the first quartile (the most asymmetric cochleae, leftmost panel), the vertical trajectory shows first a decrease and a subsequent increase (up and down arrows, “rollercoaster profile”), resulting in a distinct minimum in the second half of the first turn. This minimum was less pronounced in the second and third quartile (second and third panel). It was absent in the fourth quartile (rightmost panel), where the vertical trajectory was steadily ascending (up arrow, “sloping profile”). (**c**) Significant bivariate correlations identified from all parameters assessed. Ratio A did not show any significant correlations with the parameters measured. Those correlations that were significant (p < 0.05) are shown with reddish background.

The mean values determined with both techniques (µCT and corrosion casts) corresponded well (supplementary material, Table S1). Cochlear length can be measured in units of degree (in what follows called **angular length**) or in metric units of distance (millimeters, in what follows called **metric length**). Both the metric and the angular lengths of the cochlea were highly variable, with smallest metric length (along the lateral – outer – wall) of 36 mm and longest of 46 mm, and a difference of angular length between shortest and longest cochlea of ~200°, i.e. more than one half-turn (Fig. 2a).

Approximately half of the cochleae had the recently described ‘rollercoaster’ down-up vertical profile^24^ (Fig. 2b). The reasons for these rollercoaster profiles have hitherto been unknown. Grouping the 108 cochleae into quartiles of the ratio of how the modiolus cuts the B-axis into B_a_ and B_b_ (Fig. 1c) reveals vertical trajectories that transform from ‘rollercoaster’ to ‘sloping’ (Fig. 2b). This implies that if the modiolus intersects the B-axis asymmetrically, the cochlea is more likely to have a rollercoaster profile. ‘Rollercoaster’ cochleae frequently include a vertical jump at the point where the second turn reaches the first turn (within 450–500° ^24^). It appears as if some spatial restrictions along the B-axis would push the first and second turn closer together, and so as to fit them into the available space, deviate the first turn more basally, the second turn more apically, and caused an asymmetry along the B-axis.

Bivariate correlation analysis from the measured parameters revealed that only a subset showed significant correlations with each other (Fig. 2c). In factor analysis of those parameters (supplementary material, Fig. S1a, Table S2), angular length and B-ratio were represented in one component, and that metric length and parameters A and B were related to another component, demonstrating that there are two dissociable factors that govern the angular and the metric length. In other words, different cochleae are not simply scaled versions of some common blueprint. This suggests the usefulness of a derived factor called cochlear wrapping, a ratio between angular and metric length.

**Figure 3:**
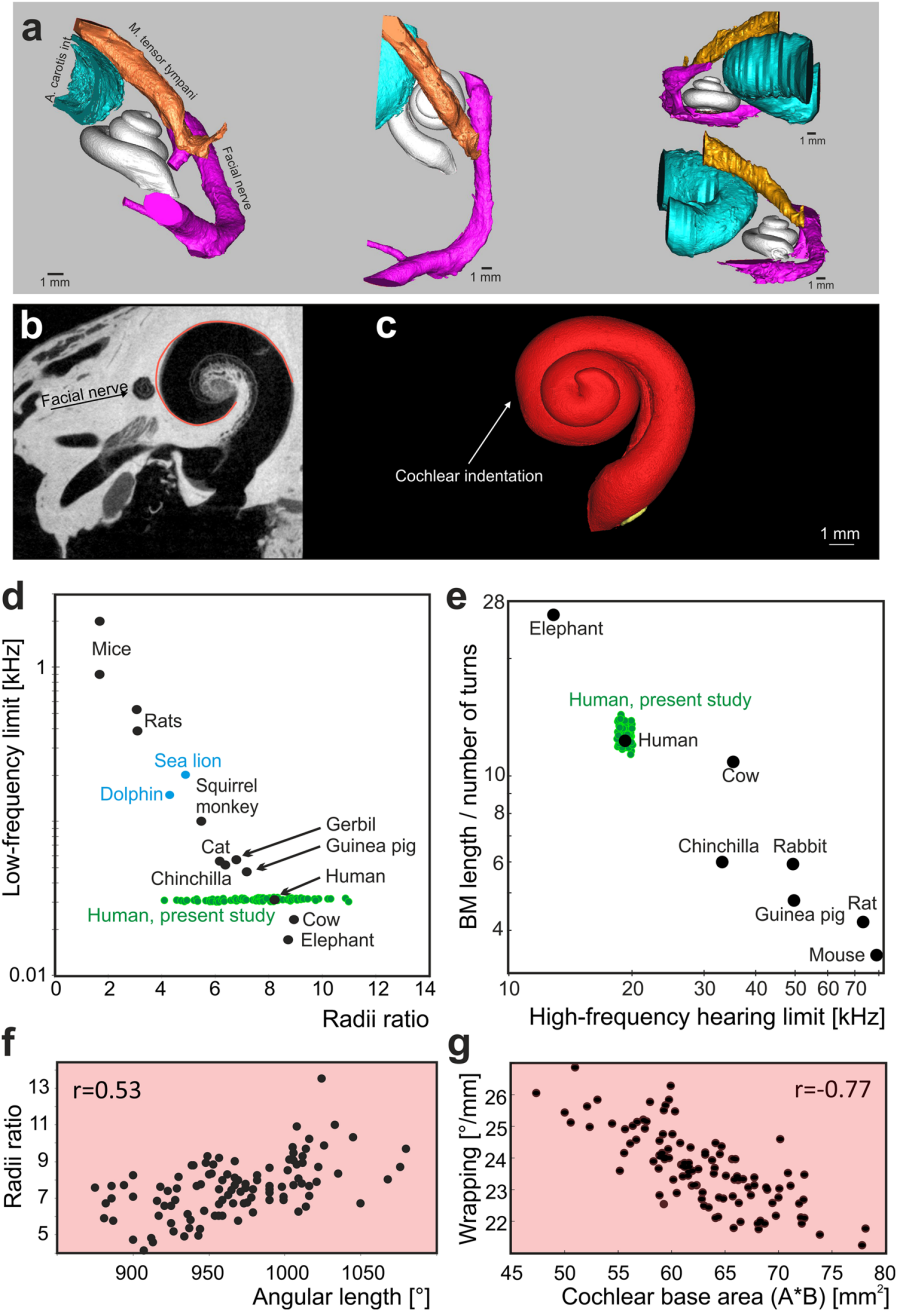
Spatial relations of the cochlea to surrounding structures that influence the cochlear shape. (**a**) µCT images revealed close proximity of internal carotid artery, facial nerve and tensor tympani muscle, shown in four different projections. (**b**) A small indentation in the cochlear shape is discernible near the place where facial nerve passes near the cochlea. (**c**) Three-dimensional reconstruction of a cochlea with cochlear indentation. (**d**) Relation of low-frequency hearing limit to radii ratio for different species (black and blue points, from^29^) as used previously to support the low-frequency focusing theory^16,17^. Interindividual variance of our human corrosion casts shown in green, with mean radii ratio of 7.57 ± 1.51. Although the mean is close to the human sample used in the previous studies, the large variance covers species from well beyond elephant down to rats. (**e**) Basilar membrane (BM) wrapping ratio (in mm/°) for different species in black (from^29^) compared to the present corrosion casts (green), approximated for basilar membrane length by data from^20^. In contrast to radii ratio, the variance in this wrapping ratio is much less than the interspecies differences. (**f**) Significant correlation of radii ratio and angular length of the cochlea indicates that the radii ratio is dependent on the number of turns of the cochlea. There was no correlation of metric length with radii ratio (not shown). (**g**) The area of the cochlear base negatively correlates with cochlear wrapping. High wrapping was observed in cochleae with small base.

Consistent with the considerations on the spatial restrictions generating the rollercoaster profiles (Fig. 2b), the cochlea is closely surrounded by several structures: the internal carotid artery, the jugular vein, the tensor tympani muscle and the facial and petrous nerve (Fig. 3a). Detailed analysis of the cochlear shape demonstrates that in the region between 270° and 300°, the shape of the cochlea sometimes deviates from it’s course, generating a small indentation (Fig. 3b,c). It is near this point that the facial nerve approaches the cochlea. Using the µCT data we measured the smallest distance between the border of the facial nerve canal and the scala tympani. In one cochlea, this distance was only 40 µm, with the average 0.23 ± 0.14 mm. Given this proximity it is plausible that the facial nerve may interfere with the growth of the cochlear spaces and be a partial cause of the variation. Confirming this, the minimal distance between the facial nerve canal and the modiolar axis correlated with B_b_ (Fig. S1b, r = 0.76, p < 0.001); thus, when the facial nerve was close to the modiolar axis, the cochlea had a smaller B_b_.

The alternative to spatial constraints shaping cochlear geometry, the functional ‘whispering gallery’ theory assumes an acoustic function^16^. It rests on the observation of a linear relation between the so-called radii ratio and the low-frequency limit of hearing in different species (Fig. 3d). Radii ratio represents the fraction of the radius of the cochlear base and the radius of the cochlear apex^16^. Here we quantified the interindividual variability in radii ratio in human corrosion casts and included it in the plot from^16^ (green dots, Fig. 3d). The range of this ratio in humans almost fully corresponded to the range of interspecies differences (Fig. 3d), contradicting the whispering gallery concept. The way in which radii ratio is defined causes angularly-longer cochleae to have a smaller radii ratio (Fig. 3f, p < 0.0001; no relation to metric length: r = 0.01; p = 0.918). Radii ratio thus relates to angular length more than to the low-frequency limit of hearing (see also^28^). On the other hand, the wrapping ratio^29^ showed interindividual variance that was substantially lower than interspecies variance (Fig. 3e). Analysis of the human data revealed wrapping to be negatively correlated with the area of the cochlear base (Fig. 3g, r = −0.77, p < 0.0001, SFig. 1). Consequently, human cochleae with smaller bases tended to be more wrapped, explaining the interindividual variability in wrapping by spatial restrictions during ontogenesis rather than a whispering gallery function. This result further supports our hypothesis that the facial nerve interferes with the growth of cochlear spaces.

**Figure 4:**
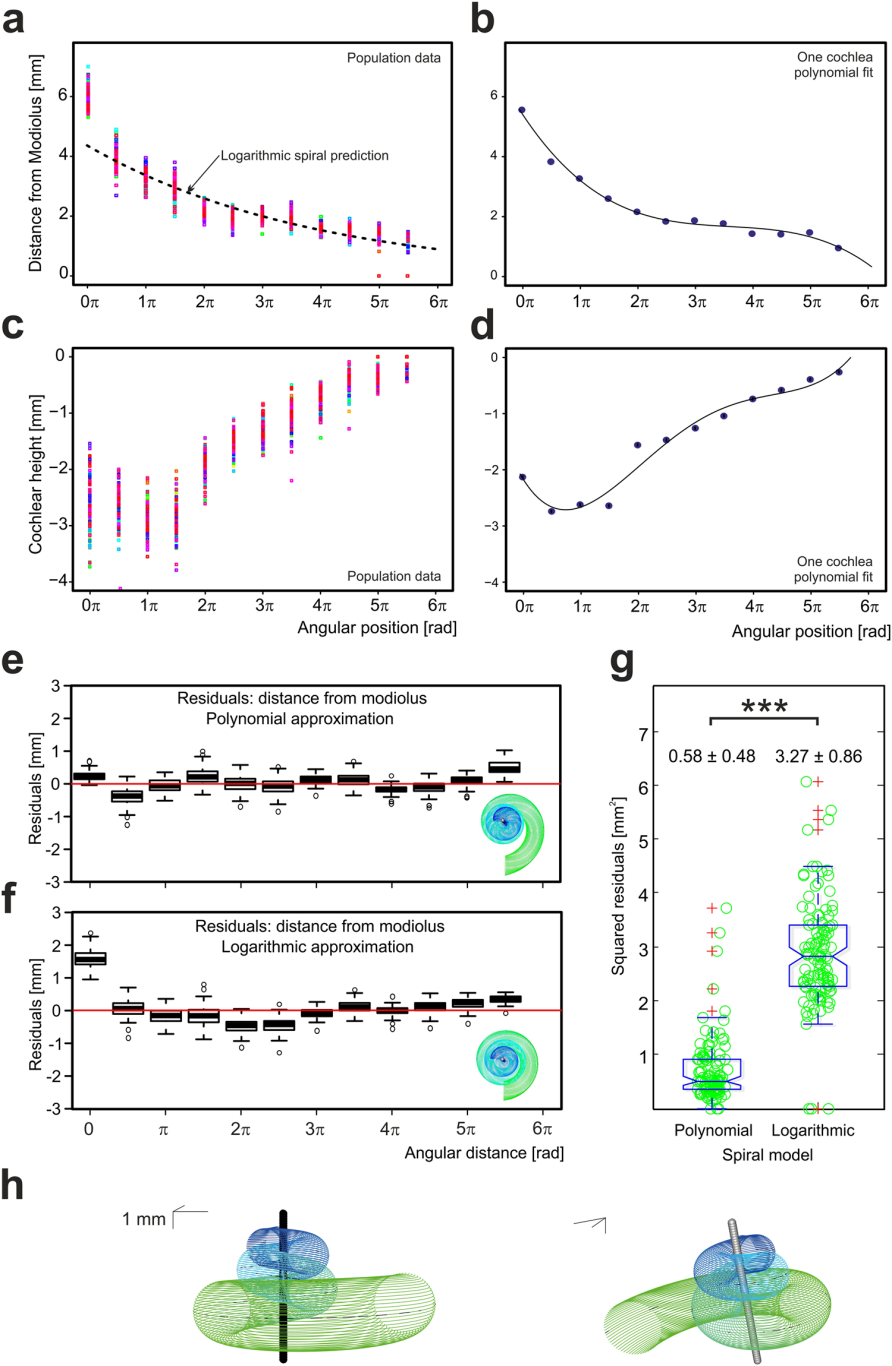
Quantified shape of the cochlea along the lateral wall and the vertical profile. (**a**) Distance of the lateral wall from the modiolus as a function of angular position, data of individual cochleae shown in different colors, logarithmic prediction shown as the dotted line. The data extensively deviate from the logarithmic prediction near 0π and 2π. (**b**) One cochlea fitted with the polynomial function. In contrast to the logarithmic function, the polynomial function fits the data of individual cochleae optimally. (**c**) Cochlear vertical position (height) as a function of angular position. Shown are all cochlea from the study, different specimen shown in different colors. (**d**) The fit of the data from one typical cochlea with a polynomial function. Here the fit was slightly weaker than in (**b**). (**e**) Results of leave-one-out technique in fitting the polynomial and logarithmic spirals to the present sample of human cochleae. Fit residuals as a function of angular position for the polynomial spiral document a good fit, with small deviations in π/2 and in the apex of the cochlea. The inset shows the typical geometry viewed from the ventral aspect very similar to the human cochlea. (**f**) Fit residuals for the logarithmic spiral. Even with the best possible fits there was a significant error at the very base, between 2π and 3π and in the apex. The inset demonstrates more tight wrapping of the basal turn in the logarithmic model compared to a real cochlea. (**g**) Summed squared residuals show a significantly and substantially weaker fit for the logarithmic spiral. Only two cochleae showed a fit to the logarithmic spiral similar to the polynomial outcomes. (**h**) Example of a 3D polynomial model when viewed from different perspectives. The lines in the inset left representing a 1 mm scale in each direction.

Next, the analytical cochlear geometry was assessed using the course of the lateral wall (Fig. 4; see also Fig. 1). Its distance from the modiolus, distance from the apex and angular length were independently fitted with mathematical analytical models. One model incorporated a standard logarithmic spiral, the other a third-degree polynomial spiral, i.e. the simplest assumption that followed from the shape of the function (Fig. 4a,b). Simple analytical model functions with few parameters were chosen to allow model prediction based only on the basic individual parameters of cochlear base width, length and their intersection by the modiolus (i.e. A_a_, A_b_, B_a_, B_b_), all measures that can be taken from conventional CT scans in a living subject.

Firstly, each of the cochleae was individually fitted (Fig. 4b) and a prediction model for the resulting model coefficients was calculated. For this purpose the fit errors were evaluated at each 90° point of the angular length of the cochlea. Secondly, the prediction errors at these points were evaluated using a leave-one-out cross-validation. For this purpose, the prediction model was repeatedly calculated for all but one of the cochleae and used to predict the form of the remainder cochlea which could then be compared to the measured data. Residual errors between prediction and the real cochlea were calculated. The logarithmic spiral generated larger residuals than the polynomial spiral for both the fitting and prediction (Fig. 4e,f). The squared residuals at the 90°-nodes (cross-validation) were summed up at the level of individual cochlea and the two models were evaluated with a paired t-test. The logarithmic spiral generated residuals that were 5.64 times larger than those generated by the the polynomial spiral (Fig. 4g, paired t-test, p = 7.7*10^−59^; mean difference of sums of squared residuals: 2.62 mm^2^, 95%-CI:[2.46 mm^2^; 2.77 mm^2^], p < 0.001).

Finally, a 3D spiral model was generated (Fig. 4h). In order to assess the cochlea’s third dimension, cochlear height relative to apex was also determined as a function of angular position (Fig. 4c,d) in a polynomial approach (degree 4, based on the apparent course of the measurements). Height prediction resulted in mean sums of squared residuals (cross-validation) of 0.88 mm^2^, 95%-CI:[0.76 mm^2^; 1.00 mm^2^]. The angular length was predicted via a linear estimation model with mean squared residuals (cross-validation) of 0.012 turns^2^, 95%-CI:[0.009 turns^2^; 0.015 turns^2^].

Based on these predictions, the metric length of predicted cochleae was calculated using the 3D models. The logarithmic 3D model performed significantly worse than the polynomial model on 90° segments (mean difference of sums of squared [segmentwise] residuals: 2.048 mm^2^, 95%-CI:[1.81 mm^2^; 2.29 mm^2^], paired t-test, p < 0.001). Both of the models predicted the metric length similarly well, with mean squared residuals (cross-validation) of 1.48 mm^2^ for the polynomial model and 1.50 mm^2^ for the logarithmic model (mean difference of squared residuals: 0.02 mm^2^, 95%-CI:[−0.08 mm^2^; 0.12 mm^2^], paired t-test, p = 0.727). Consequently, while the logarithmic model was good enough to predict the metric length of the cochlea, it failed to predict the details of the spiral shape.

The quantitative analysis of the cochlear shape thus demonstrates that the human cochlea does not have the form of a logarithmic spiral (similar to a nautilus shell), with deviation as its greatest in the cochlear base (0–90°) and at the end of the first turn (180–270°). This is also exemplified in the way the modiolus intersects the axes of the different turns: it significantly deviates from the golden ratio, particularly in the second and third turn (Fig. S2).

The polynomial model developed can be freely downloaded (in its implementation in R) from the website http://www.neuroprostheses.com/AK/CochleaModel.html.

## Discussion

The present study quantitatively analyzed cochlear form on a large set of corrosion casts at a near-microscopic resolution. The interindividual variations of the cochlear form were not compatible with a whispering gallery function of the cochlea and demonstrated that the geometry is not similar to a logarithmic spiral, as widely assumed. Instead the present data demonstrate that there are genuine interindividual differences in cochlear geometry. As the results suggest, the most likely reason for the spiral shape and the high interindividual variability are spatial restrictions within the petrous bone.

We used two sets of data in this study: µCT and corrosion casts. The good correspondence of the data (supplementary material) demonstrates that the corrosion casts are not influenced by the preparation of the casts. The only minor difference was observed in the cochlear height; here it is important to acknowledge that neither technique captures soft tissue. Corrosion casts capture only empty spaces, µCT only bony surroundings. The region in between, filled by soft tissue, is counted by neither technique. Corresponding to this interpretation, all compared measures were minimally larger in the µCT data. The difference between both methods was largest for cochlear height, likely due to soft tissue at the base and the apex of the cochlea.

The use of both techniques allowed us to analyze a large set of cochleae and at the same time analyze also structures that were not present in the corrosion casts, like facial nerve and other surrounding structures. While we tried to comply the consensus on cochlear geometry in the present study^30^ (see methods), it was not always possible (e.g. if the essential milestones were not visible in all corrosion casts). Comparing to previous literature it is important to note that this dataset reflects the length of the lateral – outer – cochlear wall, and not the length of the organ of Corti or the basilar membrane; consequently the cochlear length is larger than in studies that looked at basilar membrane length (for direct comparison, see^20^).

The observation of the high cochlear variability corresponds to previous observations^20–24,26,27,31,32^. The present study extends these reports by an exact quantitative analysis. Such quantification is of particular clinical importance since cochlear implant electrodes have been individualized in recent years and include a portfolio of different-size electrodes. The developed cochlear model allows prediction of the cochlear length (metric and angular) from measures obtainable from clinical cochlear imaging before implantation. Additional to previous suggestions of length approximation^33,34^, it provides a validated approach with assessed estimation error of the model.

The outcomes are, however, of more than practical importance. They provide a mathematical (analytical) description of the cochlear form that allows suggestions on the reasons of the form. The cochlear geometry did not fit to a simple spiral model corresponding to a nautilus. Clearly, this must be related to the shaping forces and the origins of the form itself. The nautilus shell grows from inside to outside and successively adds chambers to the shell, whereas the outside (larger) chambers are a function of the previous chambers and nutritional abundance. The cochlea, on the other hand, differentiates from the otocyst and grows in the opposite direction, i.e. from base to apex^35–37^. Thus the origins and the known constraints point to very different processes behind the shape of the cochlea and the nautilus.

In the present study we confirmed that the cochlear shape was individually highly variable. This variability was not consistent with the proposed acoustic whispering gallery function^16,17^. Due to the use of post-mortem tissue we could not correlate the outcomes with psychophysical measures of hearing. Therefore the question of functional consequences of the size and shape differences cannot be answered here (compare discussion in^24^). There are interindividual differences in density and overall number of hair cells, with longer cochleae having smaller density but slightly more hair cells^38^ that could have functional consequences. Nonetheless, the correlation of B_b_ with distance of the facial nerve and the negative correlation of the cochlear base area with wrapping rather point to spatial constraints as a source of the cochlear shape and its ontogenetic variants. Such spatial constraints are several, and therefore the shape has multifactorial anatomical causes (additional evidence in Fig. S3). In embryonic development, the neuronal structures are among the first to differentiate, and formation of cochlear spaces follows later^37,39,40^ (review in ref.^35^), making it conceivable that the position of the neural structures influences the cochlear spaces.

The present data suggest that the rollercoaster vertical profile, the asymmetry and indentation in the cochlear base, relation of angular length with base asymmetry and cochlear wrapping all result from spatial constraints in the petrous bone. The outcomes suggest that cochlear wrapping is the consequence of the balance between hearing capacity and the space available for the cochlear base. While the present analysis is largely based on correlations that cannot prove causality, the data are consistent with the cochlear form being a phylogenetic and ontogenetic consequence of efficient packing in the petrous bone, as previously hypothetized^12–15,41^. Cochlear shape is thus codetermined by genes^35,42^ and individual spatial constraints during ontogenesis.

The extensive interindividual variability shown here, and its implication for functional theories outlined in this manuscript, calls for caution when using single samples or population means in interspecies comparisons, a common approach particularly when using material from extinct species. Interindividual variability should be an obligatory component to consider in new theories.

The mathematical model developed may be used for guiding surgery and selection of individualized cochlear implants for a given subject. It requires assessment of the basic individual parameters of cochlear base width, length and their intersection by the modiolus. These parameters can be assessed using clinically available computer tomography before cochlear implantation. Such approach may further reduce implantation trauma and outcome variability in cochlear implantation.

## Materials and Methods

### Corrosion Casts

We studied 108 corrosion casts of human cochleae (59 left, 49 right) from the Hanover Human Cochlea Database. This database was established at the ENT clinics of Medical School of Hanover in 2009. It contains corrosion casts of human labyrinths from unselected temporal bones harvested in autopsies. The donors were anonymous, no biographical donor data (such as hearing status, disease, gender, age) are known.

After preparation of a concise fitted block of the bony labyrinth, specimens were dehydrated by an ascending alcohol row, then brimmed with epoxy in vacuum for 5 min. The epoxy was then allowed to stiffen for 8 hours in room temperature. Subsequently, the bony structures were then corroded in alkaline solution for 2–3 weeks until the plastic casts were completely uncovered^43^. The shrinkage factor of the epoxy resin is below 0.5%.

Imaging was performed by digital-microscopy (Keyence VHX-600, 18 Megapixel 3CCD-Camera, 30x zoom, in HDR mode) with a resolution of 12 µm/pixel. The corrosion casts were reproducibly and consistently aligned in space according to the Consensus Cochlear Coordinate System (CCCS)^30^. For this purpose an in-house developed positioning tripod allowed cross-hair-laser-assisted manipulation of the fragile casts. After free positioning in space the specimens were exactly rotated in 90°-steps for standardized imaging along perpendicular spatial axes.

Five standardized aspects were recorded for each specimen:

1. rostral: “top view” on the cochlea along the modiolar axis, a perpendicular line to the modiolar axis was aligned horizontally through the midpoint of the round-window. This view is matching the ‘plane of rotation’ of the CCCS and is equivalent to the defined radiographic projection of the ‘Cochlear View’^44^.
2. caudal: “base view” on the cochlea, exact opposite view to rostral.
3. lateral: “round-window-view” on the cochlea, perpendicular view from the vestibule on the modiolar axis, which is aligned horizontally through the midpoint of the round-window.
4. medial: “ascending spiral view” on the cochlea, exact opposite view to lateral.
5. ventral: “side view” on the cochlea, perpendicular view from ventral on the modiolar axis, which is aligned horizontally.

The dorsal view was not possible, as the positioning tripod was fixed from this side. The labeling according to the defined anatomical terms of location (rostral, caudal, lateral, medial, ventral) was used for reasons of clarity and comprehensibility. It must be kept in mind that for true anatomical aspects the modiolar axis is tilted 37.5° to lateral and slightly to anterior-inferior^45^.

The quality of the alignment was controlled by remeasuring the angle of the modiolar axis towards the cochlear base in the acquired images. The angle towards axis B lateral was 88.2 ± 3.8°; medial had the corresponding 91.8 ± 3.8°; to axis A caudal was 89.2 ± 3.6°; rostral 90.8 ± 3.6°. The individual maximum deviation from 90° found in one cochlea was 11°. The standard deviations of the data were within 4°.

120 measurement points in each of the 108 cochleae resulted in 11324 total measurements due to 818 missing values, mainly because the measurement point exceeded the given cochlea (e.g. measures at 990° were only available in cochleae that reached this angular length, in smaller cochleae these measurements were not available). Measurements of distances, angles and areas were performed with the microscope manufacturers analysis software in maximal magnification (Keyence VHX-600). Examples of the measurement scheme are shown in Fig. 1. The dimension A = A_1_ + A_2_ + … + A_7_. Similarly, B = B_1_ + B_2_ + … + B_7_. Furthermore, A_a_ = A_1_ + A_2_ + A_3_ + A_4_ and A_b_ = A_5_ + A_6_ + A_7_. Similarly, B_a_ = B_1_ + B_2_ + B_3_ + B_4_ and B_b_ = B_5_ + B_6_ + B_7_ (Fig. 1c). Ratio A was defined as A_b_/A; similarly, Ratio B was defined as B_b_/B. Measurement of cochlear length was performed with ImageJ software (Image Processing and Analysis in Java, freeware, available at http://rsbweb.nih.gov/ij/), which was calibrated for the pixel resolution. In 25 cochleae one or few measurements had to be adjusted manually due to slight damage of the corrosion cast. The cochlea outer wall length was measured in rostral view for each quadrant. Thus, the length of the cochlea corresponds to the lateral wall length. Where the calculation of the basilar membrane length was required, we used 87% of the total cochlear lateral wall length as described previously^20^.

### The model

For obtaining the model of the cochlea, the modiolar distance of the lateral wall as a function of angular distance was approximated by a logarithmic and a polynomial function (equation 2 for polynomial of 3^rd^ power based on the shape of the function shown in Fig. 3):

Logarithmic:

1 $${\hat{r}}_{\mathrm{log}}(\alpha )={a}_{0}\cdot {e}^{{\hat{a}}_{1}\cdot \alpha }\iff \,\mathrm{log}({\hat{r}}_{\mathrm{log}}(\alpha ))=\,\mathrm{log}({\hat{a}}_{0})+{\hat{a}}_{1}\cdot \alpha $$

Polynomial:

2 $${\hat{r}}_{pol}(\alpha )={\hat{b}}_{0}+{\hat{b}}_{1}\cdot \alpha +{\hat{b}}_{2}\cdot {\alpha }^{2}+{\hat{b}}_{3}\cdot {\alpha }^{3}$$

In a first step the modiolar distance was interpolated according to the two above mentioned models individually for each cochlea using a least squares regression algorithm on the linear equations. This procedure ensures an optimal conformance to the data in terms of quadratic residuals at the equidistant nodes, under the assumed model. Already at this stage the polynomial model resulted in smaller residuals and a significantly better fit. This fact may be related to the higher number of degrees of freedom in the polynomial (4) compared to the logarithmic (2) approximation but it also demonstrates the higher plausibility of the polynomial approach. The two models have been explicitly chosen for comparison since on one hand the logarithmic model has long been thought to be explanatory and has been described in the literature^6^, while on the other hand we found characteristics in the course of the modiolar distance that are specific for polynomial functions of degree at least 3 (i.e. a saddle point), indicating that a polynomial approximation as stated above may be the simplest model with promising properties.

The individual predictor values (that is, the coefficients of the model functions) were used to generate a general prediction model. To be able to extrapolate the model functions from data obtainable from conventional CTs, four parameters were used as independent variables: A_a_, A_b_, B_a_ and B_b_. Least squares regression methods were applied to model linear dependencies of the coefficients on the independent variables.

The resulting estimation models for the modiolar distance were of the form of equations 3 and 4.

3 $$\begin{array}{rcl}{\hat{r}}_{\mathrm{log}}(\alpha |{A}_{a},{B}_{a},{A}_{b},{B}_{b}) & = & \exp [{(({A}_{a},{B}_{a},{A}_{b},{B}_{b})\cdot {\hat{M}}_{log})}^{T}\cdot (\begin{array}{c}1\\ \alpha \end{array})\,]\\  & = & {e}^{\sum _{{\rm{\Theta }}\in \{1,{A}_{a},{B}_{a},{A}_{b},{B}_{b}\}}{\hat{n}}_{{\rm{\Theta }},1}\cdot {\rm{\Theta }}}\cdot {e}^{\sum _{{\rm{\Theta }}\in \{1,{A}_{a},{B}_{a},{A}_{b},{B}_{b}\}}{\hat{n}}_{{\rm{\Theta }},2}\cdot {\rm{\Theta }}\cdot \alpha }\end{array}\,$$

4 $$\begin{array}{rcl}{\hat{r}}_{pol}(\alpha |{A}_{a},{B}_{a},{A}_{b},{B}_{b}) & = & {(({A}_{a},{B}_{a},{A}_{b},{B}_{b})\cdot {\hat{M}}_{pol})}^{T}\cdot (\begin{array}{c}1\\ \alpha \\ {\alpha }^{2}\\ {\alpha }^{3}\end{array})\\  & = & \sum _{i=1}^{4}\sum _{\Theta \in \{1,{A}_{a},{B}_{a},{A}_{b},{B}_{b}\}}{\hat{m}}_{\Theta ,1}\cdot {\rm{\Theta }}\cdot {\alpha }^{i-1}\end{array}$$

Here, ${\hat{M}}_{pol}={(\hat{m})}_{{\rm{\Theta }},1}$ and ${\hat{M}}_{\mathrm{log}}={(\hat{n})}_{{\rm{\Theta }},i}$ are the coefficient matrices containing the regression estimates.

Next, in an analogous way a polynomial model for the cochlear height (based on the shape of the function shown in Fig. 3c,d, a 4^th^ power polynomial was used) was first fit to each individual cochlea and then the coefficients were related to A1, A2, B1 and B2 using least squares regression models (equation 5).

5 $$\begin{array}{rcl}{\hat{h}}_{pol}(\alpha |{A}_{a},{B}_{a},{A}_{b},{B}_{b}) & = & {(({A}_{a},{B}_{a},{A}_{b},{B}_{b})\cdot {\hat{H}}_{pol})}^{T}\cdot (\begin{array}{c}1\\ \alpha \\ {\alpha }^{2}\\ {\alpha }^{3}\\ {\alpha }^{4}\end{array})\\  & = & \sum _{i=1}^{5}\sum _{\Theta \in \{1,{A}_{a},{B}_{a},{A}_{b},{B}_{b}\}}{\hat{m}}_{\Theta ,1}\cdot {\rm{\Theta }}\cdot {\alpha }^{i-1}\end{array}$$

Finally, the number of turns (the angular distance) was measured in each cochlea. Again a linear regression was performed between the 4 cochlear base measures and the number of turns. Equation 6 allowed us to predict the overall angular length of the cochlea.

6 $${\hat{l}}_{deg}|{A}_{a},{B}_{a},{A}_{b},{B}_{b}=(1,{A}_{a},{B}_{a},{A}_{b},{B}_{b})\cdot {\hat{M}}_{deg}$$

The combination of these prediction models for modiolar distance, height and number of turns allowed us to predict the 3D form of the cochlea depending on only 4 cochlear base measures in a parametric way. It can be described in Cartesian coordinates by the equation 7:

7 $$(\begin{array}{c}\hat{x}(\alpha )\\ \hat{y}(\alpha )\\ \hat{z}(\alpha )\end{array})=(\begin{array}{c}\cos (\alpha )\cdot {\hat{r}}_{pol}(\alpha |{A}_{a},{B}_{a},{A}_{b},{B}_{b})\\ \sin (\alpha )\cdot {\hat{r}}_{pol}(\alpha |{A}_{a},{B}_{a},{A}_{b},{B}_{b})\\ {\hat{h}}_{pol}(\alpha |{A}_{a},{B}_{a},{A}_{b},{B}_{b})\end{array})\,$$

where *α* is the angular position of which the maximum value is determined by ${\hat{l}}_{{\rm{\deg }}}$. This model allows not only a comparison of different model assumptions (that is logarithmic spiral vs. polynomial spiral), but also allows to easily compute relevant calculations such as the length of cochleae by integrating over the predicted model functions. We evaluated the precision of the model estimates by means of the residuals of (i) the modiolar distance at each of the equidistant (90°) nodes, (ii) the absolute length, (iii) the number of turns and (iv) the distance from the apex (height) at each of the 90° nodes in a leave-one-out cross-validation. The mean squared residuals obtained in the cross-validation are maximum likelihood estimates for the mean squared errors for each of the validations.

Since we have valid control measurements for the absolute length based only on the ventral view of the cochlea, the height function was not accounted for in the validation. Nonetheless note that estimates for the absolute length do not differ much when incorporating or omitting the height parameters in the estimation model (mean difference of length estimation with and without height: 0.157 mm; 95%-CI: [0.154 mm; 0.161 mm]).

The technique allowed us to determine how reliable the model predicts the actual shape, provided the parameters A_a_, A_b_, B_a_, B_b_ are determined with high precision (as in the present technique).

### µCT measurements

Details of the procedure used to process the human cochleae in µCT can be found in^24^. For this study, 30 human temporal bones without any evidence of malformation were analyzed. 17 left and 13 right temporal bones were used. The bones were cut around the cochlea in blocks of approximately 3.5 × 3.5 cm, containing the outer, middle and inner ear. A standard mastoidectomy and posterior tympanotomy were performed. In order to visualize the fine structures in the cochlea, the round window (RW) membrane was opened and a small opening at the oval window was drilled. The cochlear fluid was gently removed at the round window opening^24^. This intervention allowed the image contrast between the soft tissue (basilar membrane, spiral ligament, endosteum) and the scalae to be substantially increased. Subsequently, the cochlea was wrapped with formaldehyde-immersed cotton tissue for fixation.

The temporal bones were scanned using a high-energy µCT device (Skyscan 1173, Brucker, Belgium). It included a 130 kV microfocus X-ray source within which the specimen was rotated 360 degrees between the X-ray source and camera. Rotation steps between 0.2 and 0.3 degrees were used. At each angle, an X-ray exposure was recorded on the distortion-free flat-panel sensor (resolution: 2240 × 2240 pixels, 5 Mp). To further increase the contrast, a 0.25 mm brass filter was used. In order to reduce the noise, long integration times were allowed, resulting in scan times of approximately 3 to 5 hours for each specimen; images with isotropic voxel size varying from 8–17 *µ*m were achieved in most cases, in five of these it was 36 µm.

The acquired images (TIFF format) were reconstructed using NRecon reconstruction software (Skyscan, Belgium). The reconstructed images were reoriented: the central axis (through the center of the modiolus and the helicotrema) of the cochlea was moved to the vertical direction and then the entire cochleae were reoriented based on CCCS^30^ so that individual cochleae could be directly matched and compared. Afterwards, ‘Materialise MIMICS’ software (version 14.0, Materialise, Belgium) was used to segment out the ST, Rosenthal’s canal (RC), round window (RW), and central axis of the cochlea from each of the image data sets. The precise interpolation function of the MIMICS software allowed us to semi-automatically segment between the image slices. For every 10th image slice, the region of interest was marked and subsequent images were interpolated, giving the most exact, safe, and fast segmentation. To obtain the complete geometry of the ST, segmentation was performed in both the transversal and coronal view.

### Statistical analysis

All analysis was performed in MatLab (Mathworks) and R 3.1.2 (2014-10-31). Statistical comparisons were performed using non-parametric two-tailed Wilcoxon-Mann-Whitney test and paired Wilcoxon test. Factor analysis was performed using the maximum likelihood estimate method.

## Electronic supplementary material

Supplementary material
