# Publisher Correction: Spiral Form of the Human Cochlea Results from Spatial Constraints

**DOI:** 10.1038/s41598-018-25325-8

**Published:** 2018-05-01

**Authors:** M. Pietsch, L. Aguirre Dávila, P. Erfurt, E. Avci, T. Lenarz, A. Kral

**Affiliations:** 1 Institute of AudioNeuroTechnology & Dept. of Experimental Otology, ENT Clinics, School of Medicine, Hanover Medical University, Hanover, Germany; 2 Institute of Biometry, School of Medicine, Hanover Medical University, Hanover, Germany; 3 School of Behavioral and Brain Sciences, The University of Texas, Dallas, USA

Correction to: *Scientific Reports* 10.1038/s41598-017-07795-4, published online 08 August 2017

The original version of this Article omitted an affiliation for A. Kral. The correct affiliations for A. Kral are listed below:

Institute of AudioNeuroTechnology & Dept. of Experimental Otology, ENT Clinics, School of Medicine, Hanover Medical University, Hanover, Germany.

School of Behavioral and Brain Sciences, The University of Texas, Dallas, USA.

In addition, this Article omitted an affiliation for T. Lenarz. The correct affiliation for T. Lenarz is listed below:

Institute of AudioNeuroTechnology & Dept. of Experimental Otology, ENT Clinics, School of Medicine, Hanover Medical University, Hanover, Germany.

