# Supplementary material for: Spiral Form of the Human Cochlea Results from Spatial Constraints
Source: Sci Rep. 2017 Aug 8;7:7500. doi: 10.1038/s41598-017-07795-4 (PMC5548794; doi:10.1038/s41598-017-07795-4)
Supplement: Supplementary file 1 — Supplementary material [file 41598_2017_7795_MOESM1_ESM.doc]

**Supplementary material**

**Spiral Form of the Human Cochlea Results from Spatial Constraints**

*M. Pietsch^1^, L. Aguirre Dávila^2^, P. Erfurt,^1^ E. Avci^1^,*

*T. Lenarz^1^ and A. Kral^1^,*^3, CA^

^1^ Institute of AudioNeuroTechnology &

Dept. of Experimental Otology, ENT Clinics

School of Medicine, Hannover Medical University

Germany

^2^ Institute of Biostatistics

School of Medicine, Hannover Medical University

Germany

^3^ School of Behavioral and Brain Sciences

The University of Texas, Dallas, USA


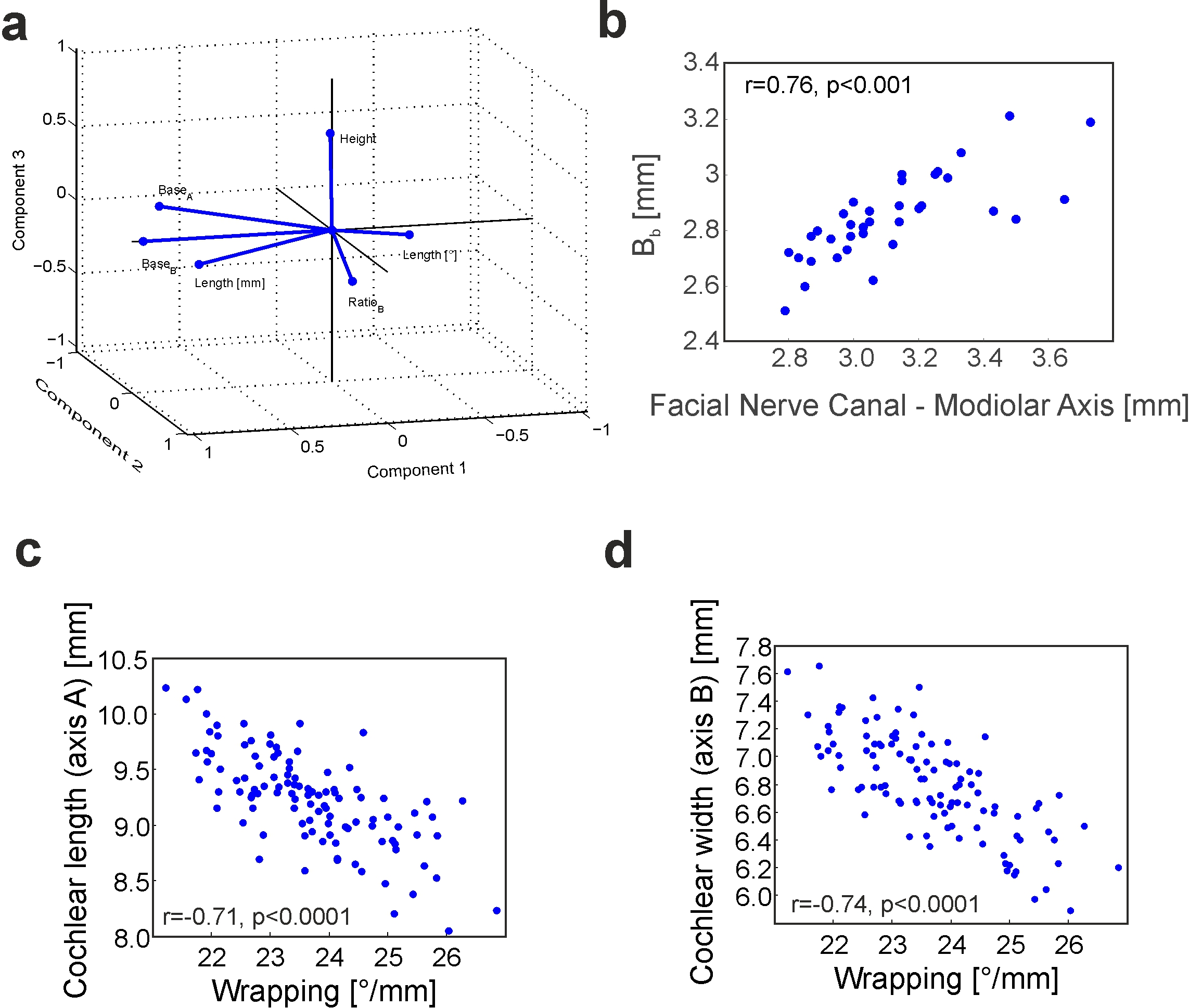


Fig. S1: Factor analysis of the main interdependent factors reveals that angular and metric length are different factors and their ratio, cochlear wrapping, correlates with cochlear base measures. a: Multivariate (factor) analysis of basic cochlear measures relate the metric length (in mm) and angular length to different components. b: Correlation of B_b_ with distance of facial nerve from the modiolus, calculated in the µCT data where position of facial nerve could be resolved (n=30). c,d: Bivariate correlations of the derived variable wrapping with cochlear length (c) and cochlear width (d) with base area (comp. Fig. 3). These correlations reveal that the smaller the cochlear base dimensions, the larger the wrapping (°/mm).


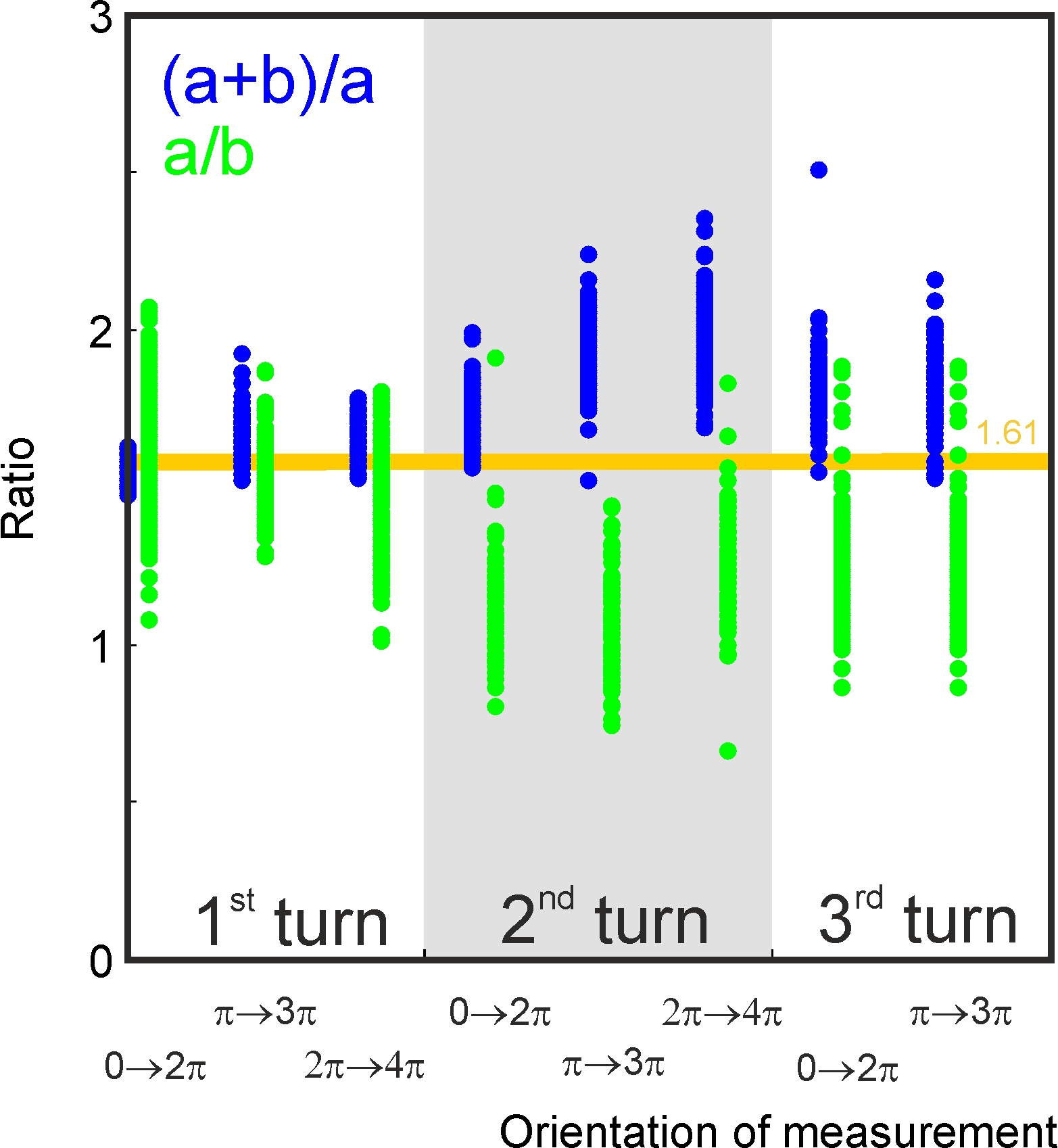


Fig. S2: Ratio in which modiolus intersects the axis of the cochlea, measured at two different planes (perpendicular to each other and corresponding to the planes of base A and base B) along the three turns of the cochlea. ‘a’ corresponds to the longer dimension of the axis, ‘b’ to the shorter dimension of the axis. Calculated are two different alternative proportions used in calculating the golden ratio (indicated by the color of the data points and formula). Although in the 1^st^ turn the data could correspond to the golden ratio, in the 2^nd^ and 3^rd^ turn the data deviate significantly from it. These data further demonstrate the irregular shape of the cochlea.


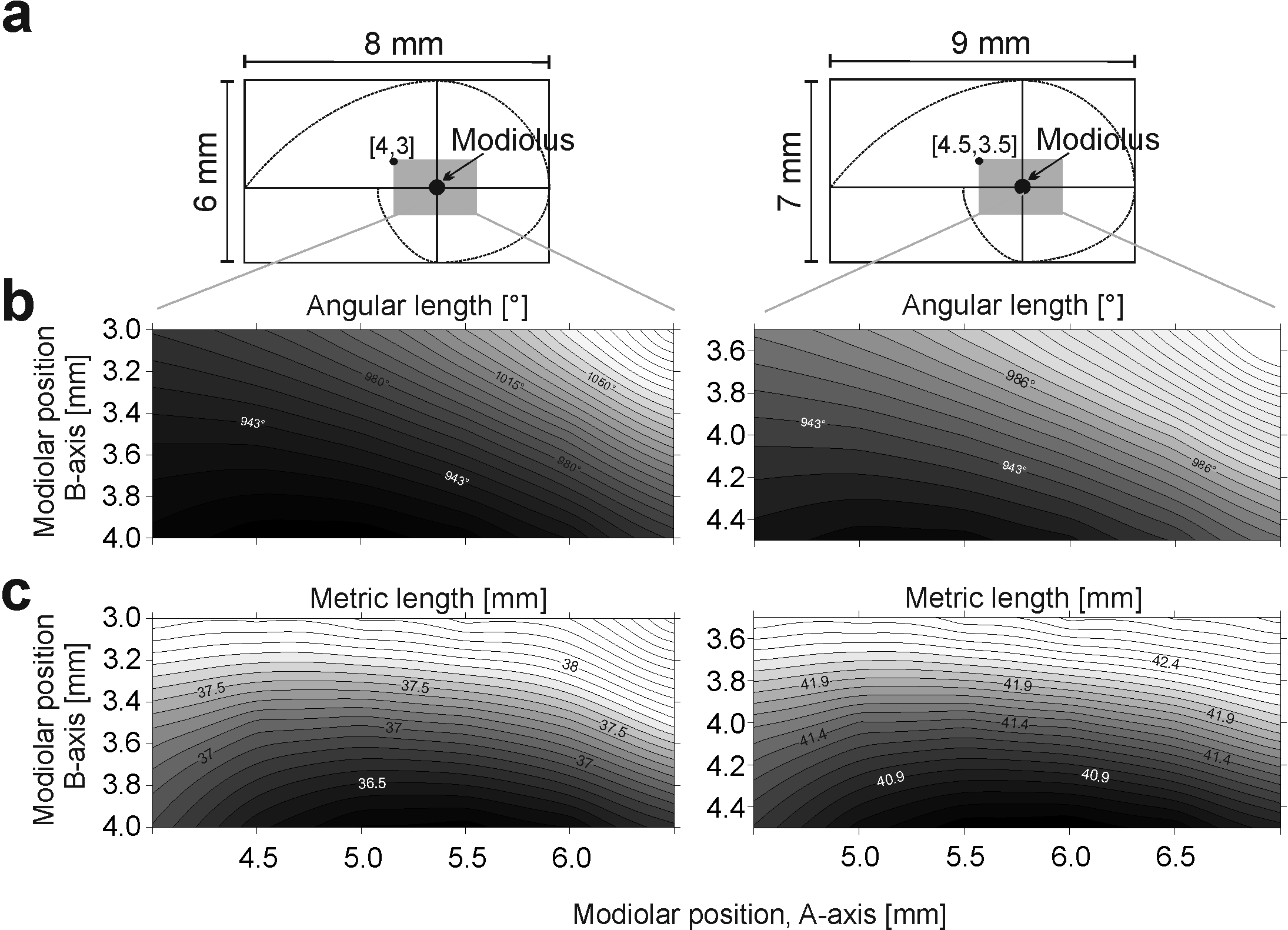


Fig. S3: Model predictions on the relation of A, B, cochlear angular length and width document multifactorial relations. Assuming that the model reflects the shape of the human cochleae well, we used it to systematically explore the shapes of these cochleae, when continuously varying A- and B-ratios and keeping the size of the cochlear base constant (a). Using this approach the model predicted interdependence and angularly-longer cochleae if the B-ratio was symmetric (b, left panel, when the modiolar axis was close to 3 mm in the B dimension) and the A-ratio asymmetric. Note that those cochleae with smaller basal dimensions resulted in greater angular length, corresponding to measurements in Fig. 3g. Angular length was, however, not dependent on the A-ratio alone (r=0.07, p=0.45). Thus, in the absence of the stronger effect of an asymmetry in the B-dimension the effect along the A-dimension may occur and increase angular length. Combined this suggests an anatomic influence (probably the carotid artery) that may ‘force’ the cochlea to initiate the wrapping process earlier (resulting in greater angular length with the same metric length). The presence of an asymmetric B-ratio (influenced by facial nerve) has, however, a dominating influence on both metric and angular length and may obscure this weak effect. c: Metric length appears more dependent on B than on A, consistent with the outcome that angular and metric length are different measures of cochlear shape.

**Supplementary tables:**


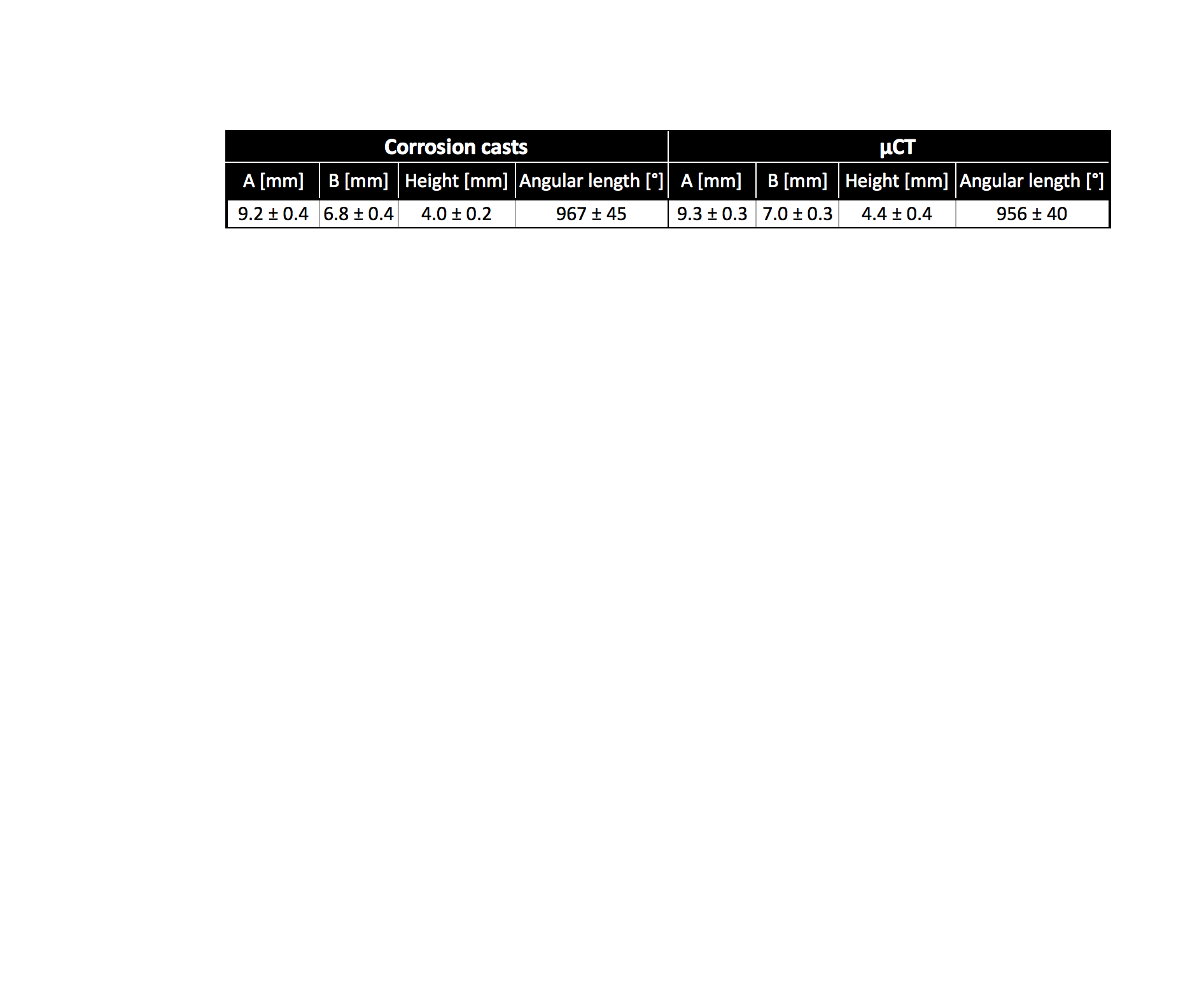


Table S1: Correspondence of corrosion cast data and µCT data. Results of corrosion casts and µCT data correspond well, considering that corrosion casts represent a negative image (cochlear spaces) whereas µCT represents the positive image (the bony walls). Soft tissue is not counted in either method and explains the small differences in the measurements. µCT tends to overestimate the size of the scalae.


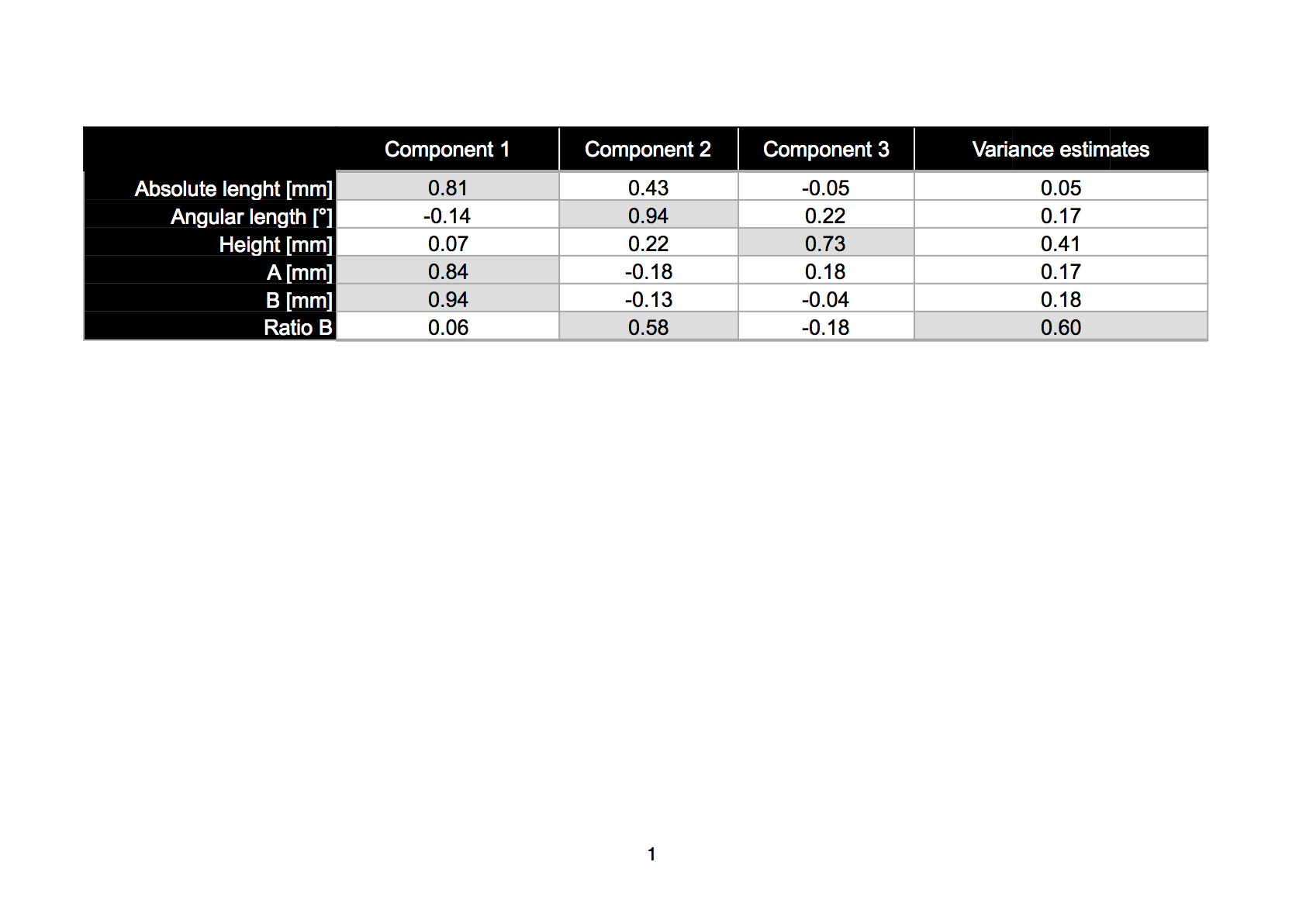


Table S2: Three main components of the factor analysis of the five parameters that were intercorrelated in bivariate analysis. The data revealed that despite bivariate correlation of angular and metric lengths, these two parameters were separated into two different perpendicular components.
